# Design preferences and cognitive styles: experimentation by automated website synthesis

**DOI:** 10.1186/1759-4499-4-2

**Published:** 2012-06-29

**Authors:** Siu-wai Leung, John Lee, Chris Johnson, David Robertson

**Affiliations:** 1 School of Informatics, University of Edinburgh, Edinburgh EH8 9AB, UK; 2 State Key Lab QRCM and ICMS, University of Macau, Taipa, Macao, China; 3 Department of Computing Science, University of Glasgow, Glasgow G12 8QQ, UK

## Abstract

**Background:**

This article aims to demonstrate computational synthesis of Web-based experiments in undertaking experimentation on relationships among the participants' design preference, rationale, and cognitive test performance. The exemplified experiments were computationally synthesised, including the websites as materials, experiment protocols as methods, and cognitive tests as protocol modules. This work also exemplifies the use of a website synthesiser as an essential instrument enabling the participants to explore different possible designs, which were generated on the fly, before selection of preferred designs.

**Methods:**

The participants were given interactive tree and table generators so that they could explore some different ways of presenting causality information in tables and trees as the visualisation formats. The participants gave their preference ratings for the available designs, as well as their rationale (criteria) for their design decisions. The participants were also asked to take four cognitive tests, which focus on the aspects of visualisation and analogy-making. The relationships among preference ratings, rationale, and the results of cognitive tests were analysed by conservative non-parametric statistics including Wilcoxon test, Krustal-Wallis test, and Kendall correlation.

**Results:**

In the test, 41 of the total 64 participants preferred graphical (tree-form) to tabular presentation. Despite the popular preference for graphical presentation, the given tabular presentation was generally rated to be easier than graphical presentation to interpret, especially by those who were scored lower in the visualization and analogy-making tests.

**Conclusions:**

This piece of evidence helps generate a hypothesis that design preferences are related to specific cognitive abilities. Without the use of computational synthesis, the experiment setup and scientific results would be impractical to obtain.

## Background

The manner of external representation (or presentation) could affect our way of working with the internal representation (mentally) and our understanding of the information [1], e.g. in cockpit information displays for aviation [2], but few results on graphical external representation can be generalised [3].

One problem affecting all websites is that there is no reliable, general and abstract method for predicting the effect of presentation rhetorics and modality on understanding of the information. To improve knowledge communication, we should investigate how sensitive people might be to differences in the way we construct our websites. It would be useful to conduct experiments quickly to compare different models of interpretation of information of a specific domain in particular cases. There may be certain styles of presentation or navigation that are generally demanded by users and can either hinder or support users' ability to interpret the information.

Tabular and graphical representations are common in constructing visual arguments [4] and presenting relational data (especially quantitative data) [5]. Visualisation of aviation accident events generally use causal trees to represent the causal relations but there are few empirical studies on both preference and perception of causality visualisation. Specifically, we investigate users' preferences for information visualisation styles and their perception of causality as required by aviation accident reporting. As the Web is one of the main channels for publishing information of aviation accidents, it is desirable to know about how people would prefer the causal relations in accident events to be presented in a website and how they perceive this causality. The user preference data are useful in the design and re-design of websites. To elicit preferences from people, we provide multiple designs for selection and study the rationale of their design decisions. Automated website synthesis saves time and effort in building websites for such designs. Few models and theories are available to address computational website design. However, if we view websites as a form of information visualisation, we can borrow some findings from automated diagram design [6] to serve as our experiment hypotheses. Some systems for automated diagram design have incorporated text to enhance user understanding of graphical visualisation [7]. This kind of multimodal visualisation should be applicable to website design. Expressiveness and effectiveness of graphical languages as proposed by Mackinlay [8] have been influential to diagram visualisation models, including source information characteristics [9], user-defined task specification [10], and user-defined layout preferences [7] for automated diagram design.

In general, we would like to see if people would prefer different representations to display the same information. In particular, this study aims to elicit preferences of designers (and users) about visualisation patterns, particularly the preferences for tables and trees in visualising causality information. In this case, we select trees and tables as the options for selection by users. Tree representations are commonly used to graphically represent causality in printed documents. The causal relations are normally represented by arrows or lines connecting causes and effects.

If people do prefer a representation, it would be interesting to see what rationale or criteria contribute to their preferences. We categorised common rationale/criteria mentioned in website design textbooks [11,12]:

• easy to learn: the users do not need much time and effort to understand how it works;

• more visual: the users can understand through graphical illustrations;

• more informative: the users can know more details;

• more scalable: fewer changes are needed to handle more massive information;

• more features represented: less characteristics (important information) are left out;

• more suggestive: the users can understand without much guessing; and

• more flexible: suitable for use in different situations.

As these seven rationales may not cover all possible rationales that are crucial to any particular preference, the experiment participants were asked (unprompted) for their rationale before seeing these seven rationales and then they were asked (prompted) to identify if any of these rationales were similar to their own rationales. They were also asked if any of their rationales was not covered by these seven rationales.

Software designs should reduce users' cognitive load [13]. We hypothesise that participants prefer one design to other designs partially because the preferred design suits their cognitive abilities. If this is true, the cognitive abilities of the participants should be related to their preferences. The relationships among preferences of trees or tables, the cognitive test results of the participants, and rationale for their preferences were studied in this experiment. As it is impossible to test numerous cognitive factors in a single experiment, the participants were only tested on cognitive styles/abilities of visualisation and analogy-making, which we guessed were related to visual representations.

The main objectives of this experiment are as follows:

• To see if there is any different preference for tables or trees in representing the given information;

• To see if different preferences are based on different priority in criteria/rationale;

• To see if the preferences are related to the cognitive test results; and

• To see if the importance ratings of design criteria/rationale are related to cognitive test results.

## Methods

### Participants

Sixty four students from the University of Edinburgh participated in the experiment and received cash (GBP 10) as a reward. They were randomly assigned to one of the two groups according to a pre-generated random sequence. Each group had 32 participants. The treatments of these two groups differ in the order of using table and tree design generators (or simply called designers). All of the participants had the computer skills for browsing websites. The experiment took about 1.5 hours for each participant. No strict time limit was enforced for tasks except cognitive tests, for which data were automatically collected.

### Web pages

Computational website synthesis provided basic facilities for generating websites and their functional components such as menus and breadcrumbs. We mapped the information content items to appropriate components in specifications. Presentation of accident event (content) information in visualisation formats (tabular cells or graphical trees) required mappings of attributes between the content information and visualisation formats. One of the major factors affecting the mapping decision was designers' (and users') preferences.

**Figure 1:**
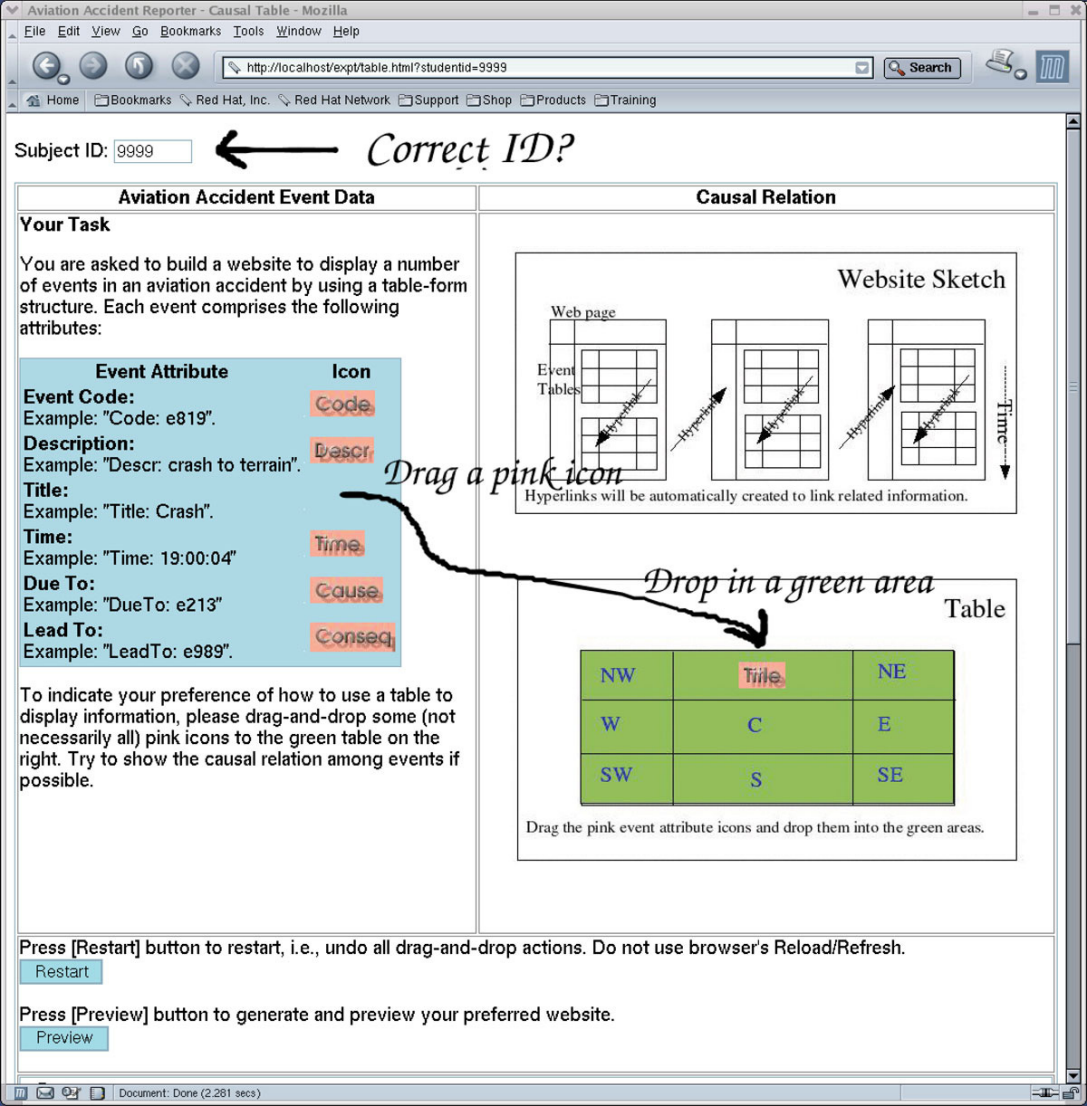
**Exploring options for tabular representation**.

**Figure 2:**
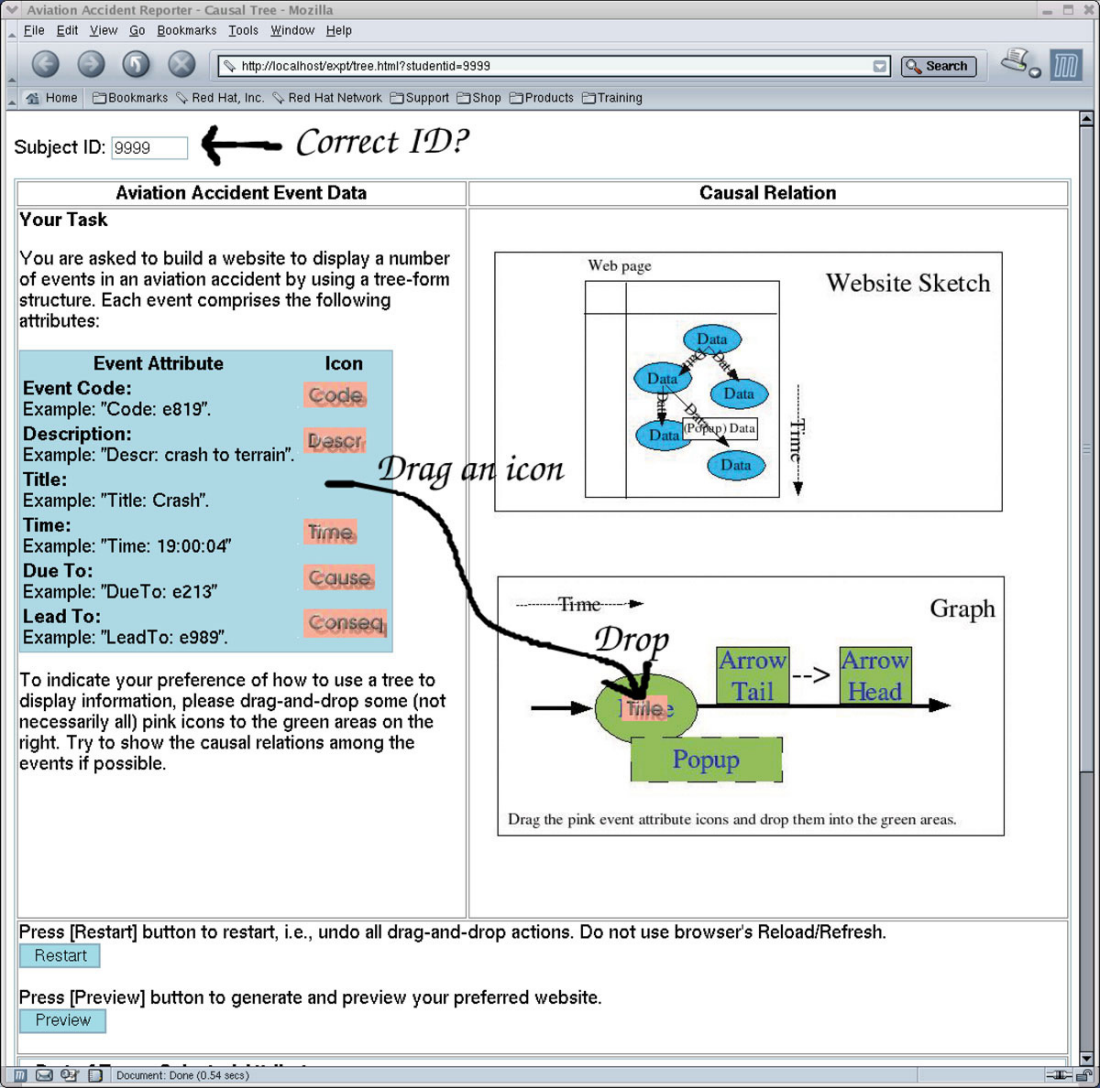
**Exploring options for tree form representation**.

Our approach to eliciting the designers' preference is to let the designers explore the available options and then decide which option is the one they prefer. For doing this, we gave the information of the customised images (representing specific pieces of information) to a home-made drag-and-drop web component as parameters so that the users could design their preferred tables (Figure 1) and trees (Figure 2) by dragging and dropping the representational images (one at a time) in a position relative to any specific tiny (1 × 1 pixels) image dot (invisible). A drop of the representational image was successful only if it was dropped within an area of a predefined radius to the anchor image. If a representational image was dropped outside the specified area, then it would return to its original position. The repositioning would be activated whenever there was a change (e.g. change of window size which affects the relative positions of images). A piece of JavaScript code was generated to feed these parameters to a JavaScript component for image drag-and-drop management. This approach was simpler (lightweight) than many other approaches which used sophisticated (heavyweight) Java Applets or Flash objects to provide drag-and-drop functions.

### Data collection

All input from the participants were collected by standard HTML forms and CGI (common gateway interface) scripts, which were generated from simpler specifications for defining variables and variable types (e.g. multi-answers or long text) and special webpage elements. Subsequent minor modifications to the generated questionnaire forms were only for cosmetic purposes.

### Tree and table generators

The trees and tables were generated on-the-fly and viewable in a separate window of the web browser together with other basic website navigation facilities, e.g. menus and hyperlinks. The tables were generated as standard HTML tables while the trees were generated as DOT diagram specifications for final image rendering by GraphViz on the server side before sending to the client side.

### Tasks

Each participant filled in a pre-experiment questionnaire which collected background information such as their familiarity with the Web and aviation operations. Then the participants used table and tree designers to express their preferences between tables and trees in representing a given structure of causality information. The order of using table and tree designers were randomly assigned. Participants assigned to Group A used the tree designer first and then the table designer while those in Group B used the table designer first and then the tree designer. In the designers, the participants used a customised drag-and-drop facility to design their preferred table and tree patterns. The participants submitted their preferred visualisation patterns as tables or trees. Their rationale for their preferred visualisation pattern were collected by a two-part post-experiment questionnaire. Part A of the questionnaire collected their rationale as the open-ended answers and their preference ratings of table and tree visualisation. Part B of the questionnaire collected their ratings of the importance against seven common preference criteria/rationale. The participants also took timed cognitive tests, including two (paper folding and surface development) visualisation tests and two (visual and verbal) analogy-making tests. The visualisation tests were licensed from the Educational Testing Services of the USA, as provided in a kit of factor-referenced cognitive tests [14]. The visual analogy puzzles were the same as those selected by Thomas Evans [15] in his study of visual analogy problems. The verbal analogy test was the sample questions of the Miller Analogy Test (MAT).

### Data analysis

Variables included (1) preferences of tables or trees, (2) importance ratings of preference criteria/rationale, and (3) results of cognitive tests. Data were presented in medians, means, and standard deviations (SD) for both parametric and non-parametric analyses, although only conservative non-parametric Wilcoxon test, Krustal-Wallis test, and Kendall rank correlation test were reported in this article. Both parametric (e.g. t-test and ANOVA) and non-parametric tests were run using R statistical software [16] and its *μ*Stat package. In multiple comparison, P values was adjusted by Bonferroni correction. P values less than 0.05 were considered statistically significant. In statistical tables, single asterisks (*) indicated P < 0.05 and double asterisks (**) indicated P < 0.01.

## Results

### Background of participants

There was no significant difference in any background variable between two groups of participants as measured by the pre-experiment questionnaire and analysed by Student's t-test and Wilcoxon test.

### Preferences

Preferences were obtained from the preference ratings about participants' preferences between tables and trees as the representation of the given information. The participants rated the strength of their preferences as:

• strong table preference,

• moderate table preference,

• marginal table preference,

• marginal tree preference,

• moderate tree preference, and

• strong tree preference.

Based on the types of preferred representations, binary classification gives two categories of participants: table preferrers and tree preferrers. The number of participants under these classifications of preferences were counted as shown in Table 1. There were 23 participants preferred tables and 41 participants preferred trees.

**Table 1:** Preference of table and tree representations

Preferences	Class	No. of participants
Strong table	Table	3
Moderate table	Table	14
Marginal table	Table	6
Marginal tree	Tree	6
Moderate tree	Tree	15
Strong tree	Tree	20

### Rationales

Seven rationales/criteria were given to the participants to rank using the numbers 1-7. Rank 1 is the most important rationale or criteria in their preference decision. Rank 7 is the least important one. The summary statistics including median, mean, and standard deviation (SD) of the overall ranking on the common design criteria/rationale is shown in Table 2. This overall result indicates that the participants found "more informative" and "easier to learn" as the most important two rationales for their preference decisions. "More Visual" and "more suggestive" were moderately important.

**Table 2:** Rationales for preferences

Rationale	Median	Mean	SD
Easier to learn	3	3.09	2.01
More visual	4	4.06	1.78
More informative	2	2.78	1.86
More scalable	5	4.90	1.42
More features	5	4.86	1.90
More suggestive	4	3.98	2.00
More flexible	5	4.86	1.78

### Cognitive tests

The summary statistics of the test results are as shown in Table 3. The test of surface development visualisation seemed to be difficult to some participants. The median of the result was 0 and its standard deviation was high.

**Table 3:** Results of cognitive tests

Test	Median	Mean	SD
Paper folding	5	5.33	2.24
Surface development	0	8.59	11.14
Visual analogy	17	15.89	3.88
Verbal analogy	8	9.97	7.49

### Preferences and rationales

As shown in Table 4 the differences in the rankings of the rationale "easier to learn" among different participants with different strengths of preferences were found statistically significant by using the Krustal-Wallis test. The difference between table preferrers' and tree preferrers' rankings of the rationale "easier to learn" was highly statistically significant as indicated by the Wilcoxon test. The significant difference related to the rationale "more suggestive" was only observed in binary preferences, not in the original classification of preferences and their strengths.

**Table 4:** Relationship between preferences and rationales

Rationale	Original preferences			Binary preferences		
	*χ*^2^	*P*		*χ*^2^	*P*	
Easier to learn	11.88	0.037	*	9.22	0.002	**
More visual	7.37	0.195		1.60	0.207	
More informative	5.10	0.404		1.25	0.264	
More scalable	5.07	0.408		1.48	0.225	
More features	6.90	0.228		3.84	0.050	
More suggestive	7.37	0.194		5.69	0.017	*
More flexible	8.57	0.127		0.23	0.633	

Table 5 showed the median rankings of rationale for preferences. Table preferrers found the criterion "easier to learn" to the most important rationale while the tree preferrers did not.

**Table 5:** Ranking of rationales

Rationale	Table Preferers			Tree Preferers		
	Median	Mean	SD	Median	Mean	SD
Easier to learn	1	2.13	1.74	4	3.63	1.96
More visual	3	3.74	1.79	4	4.24	1.79
More informative	3	3.04	1.99	2	2.63	1.80
More scalable	4	4.65	1.27	5	5.00	1.50
More features	6	5.65	0.98	5	4.42	2.14
More suggestive	5	4.78	1.86	3	3.54	1.94
More flexible	5	5.04	1.64	5	4.76	1.87

### Preferences and cognitive tests

The relationship between the results of visual analogy test and preferences (and binary preferences) was highly significant (P < 0.01) according to the Kruskal-Wallis test (and Wilcoxon test), as shown in Table 6. It was highly significant (P < 0.01) that the participants who performed better in the visual analogy test preferred trees (Table 7).

**Table 6:** Relationship between cognitive test results and preferences

Test	Original preference			Binary preference		
	*χ*^2^	*P*		*χ*^2^	*P*	
Paper folding	9.16	0.103		5.10	0.024	*
Surface development	6.09	0.298		0.15	0.697	
Visual analogy	16.36	0.006	**	13.50	0.000	**
Verbal analogy	3.95	0.557		1.14	0.286	

**Table 7:** Comparison of cognitive test results between different groups of preferrers

Test	Table Preferers			Tree Preferers		
	Median	Mean	SD	Median	Mean	SD
Paper folding*	4	4.57	2.04	5	5.76	2.26
Surface development	0	7.61	11.16	0	9.15	11.22
Visual analogy**	16	13.57	5.66	17	17.2	1.14
Verbal analogy	9	9.96	6.47	7	9.98	8.08

### Rationale and cognitive tests

The correlation between the importance rankings of rationale ("easier to learn" and and the result rankings of cognitive tests are statistically significant (Table 8) according to Kendall's rank correlation test. The

**Table 8:** Correlation between rationales and cognitive test results

Tests	Easier to learn			More suggestive		
	*τ*	*P*		*τ*	*P*	
Paper folding	0.203	0.039	*	-0.050	0.608	
Surface development	0.173	0.088		-0.070	0.484	
Visual analogy	0.263	0.008	**	-0.201	0.040	*
Verbal analogy	0.006	0.948		0.058	0.532	

statistically significant rank correlations coefficient (*τ*) ranged between around 0.201 - 0.263, which are only low to moderate in strength.

## Discussion

This study used website synthesis to construct an experimental apparatus for Lab-on-the-Web. Using this approach we found a significant relationship among the participants' preferences, rationale, and cognitive ability test results. Most (41 out of 64) participants claimed themselves to be tree preferrers. The other participants (23 out of 64) said they preferred tables. Both table preferrers and tree preferrers found their preferred representations (tables or trees) more informative, without significant difference in rankings of this rationale. As the task given to the participants is to represent information, it is not surprising that the rationale "more informative" was one of the most important rationale. In further studies, it would be interesting to test whether the participants find the same rationale justifiable for their preferences when they are given different goals or under different conditions. This might give us more insights into how different preferrers perceive information.

To table preferrers, the rationale "easier to learn" was more important than "more informative". The rationale "easier to learn" was ranked as the most important by 13 out of 23 table preferrers, but not so important by tree preferrers. Tree preferrers ranked the rationale "more suggestive" significantly higher than the table preferrers did. To table preferrers, tables seemed to be easier to learn than trees. To tree preferrers, trees were more suggestive than tables. These discrepancies in rationale ranking indicate a perception difference between different preferrers in perceiving the given tables and trees.

Tree preferrers performed better than table preferrers in some tests of cognitive factors, particularly the paper folding visualisation test and visual analogy test. There was no significant difference between table preferrers and tree preferrers in their performance in other cognitive tests including the surface development visualisation test and the verbal analogy test. It is plausible that the interpretation of tree representations requires specific cognitive capabilities such as visualisation and visual analogy-making; thus, those who do not feel comfortable with these tasks would prefer tables and highly rank the rationale "easier to learn" for their table preference.

Among all participants, there is a statistically significant low-to-moderate rank correlation between the rankings of the rationale "easier to learn" and the results of visual analogy test and paper folding visualisation test. The low-to-moderate rank correlation indicates that the participants who performed better in such two cognitive tests ranked the rationale "easier to learn" to be less important. At similar correlation strength, the participants who performed better in visual analogy test ranked the rationale "more suggestive" to be more important. It seems that the visualisation and visual analogy-making abilities of the participants might play a role in their preferences for tables and trees. Possibly (although we cannot prove this), table preferrers lack sufficient cognitive capability to interpret graphical representations like trees; thus, they prefer tables as they are easier to learn. Tree preferrers would feel more comfortable in making visual analogy and find graphical representations like trees suggestive. As indicated by the low to moderate strengths of correlation in these results, it is probable that other factors (and other cognitive factors) may be also relevant to the participants' preferences. Further studies are required to delineate these relationships.

This experiment also demonstrated the technological significance of computational synthesis in enabling scientific experiments. This experiment re-used a website synthesiser to generate websites on the fly so that the designers/users can explore the design space. Without using a website synthesiser, this experiment would not be possible. Other than computational synthesis, the most relevant tool is Web content management system but it does not meet the requirement of this experiment. Apart from its high cost, there is no Web content management system so flexible as our our website synthesiser in accepting information mappings. This makes Web content management systems inapplicable to our experiment. Thus, computational synthesis is the only available solution for this experiment although computational synthesis following definite patterns would limit the variability of graphical presentation. To enrich the variability and functionality of graphical presentation, we would consider using web design patterns available in Web 2.0 and HTML5 to enhance further studies.

## Conclusions

The experiment reported in this article found significant relationships between the participants' design preference, rationale, and cognitive test performance. This work also exemplifies the use of a website synthesiser as an essential instrument enabling the participants to explore different possible designs, which were generated on the fly, before they selected their preferred designs. In the tested sample, more people prefer graphical to tabular presentation. Despite the high preference for graphical presentation, the given tabular presentation was generally rated to be easier than graphical presentation to interpret, especially for those who score below average in the visualisation and analogy-making tests. This piece of evidence helps generate a hypothesis that design preferences are related to specific cognitive abilities. Without the use of computational synthesis, the experiment setup and scientific results would be impractical to obtain.

## Authors' contributions

DR and SL conceived this study. JL and CJ gave technical advice about web design and contribute knowledge in aviation accident domains. SL conducted the experiment and drafted the manuscript. DR and JL revised the manuscript. All authors read and approved the final manuscript.
